# Addition of High Acyl Gellan Gum to Low Acyl Gellan Gum Enables the Blends 3D Bioprintable

**DOI:** 10.3390/gels8040199

**Published:** 2022-03-23

**Authors:** Ashwini Rahul Akkineni, Bilge Sen Elci, Anja Lode, Michael Gelinsky

**Affiliations:** Centre for Translational Bone, Joint and Soft Tissue Research, University Hospital Carl Gustav Carus, Faculty of Medicine of Technische Universität Dresden, Fetscherstr. 74, 01307 Dresden, Germany; bilge.elci@epfl.ch (B.S.E.); anja.lode@tu-dresden.de (A.L.); michael.gelinsky@tu-dresden.de (M.G.)

**Keywords:** gellan gum, bioink, 3D printing, additive manufacturing, microextrusion, direct ink writing, biofabrifaction

## Abstract

Long-term stability of gellan gum (GG) at physiological conditions is expected, as very low concentration of divalent ions are required for crosslinking, as compared to alginate—which is extensively used for tissue engineering (TE) applications. Hence, GG is proposed as an ideal candidate to substitute alginate for TE. Deacylated (low acyl; LA) GG forms brittle gels, thus only low concentrations were used for cell encapsulation, whereas acylated (high acyl; HA) GG forms weak/soft gels. 3D bioprinting using pure LAGG or HAGG is not possible owing to their rheological properties. Here, we report development and characterization of bioprintable blends of LAGG and HAGG. Increase in HAGG in the blends improved shear recovery and shape fidelity of printed scaffolds. Low volumetric swelling observed in cell culture conditions over 14 days indicates stability. Volumetric scaffolds were successfully printed and their mechanical properties were determined by uniaxial compressive testing. Mesenchymal stem cells bioprinted in blends of 3% LAGG and 3% HAGG survived the printing process showing >80% viability; a gradual decrease in cell numbers was observed over 21 days of culture. However, exploiting intrinsic advantages of 3D bioprinting, LAGG/HAGG blends open up numerous possibilities to improve and/or tailor various aspects required for TE.

## 1. Introduction

Development of suitable biopolymers has always been the crux for developing regenerative and tissue engineering (TE) approaches. Many biopolymers, both synthetic and natural, used in TE have been developed to cater specific needs such as encapsulation, proliferation, differentiation of various cells, stability of constructs, and controlled delivery of biological components. Among the naturally derived biopolymers, alginate has been extensively studied and employed, due to its easy availability, biocompatibility, and processability for TE applications. Collagen, gelatin, fibrin, chitosan, gellan gum (GG), hyaluronic acid, etc. have also been widely employed for TE applications. The basic criteria for any biopolymer hydrogels to be used for TE is the ability to aid encapsulated cells to perform their functions and to provide essential structural support when implanted. Additive manufacturing (AM) methods like inkjet printing and extrusion printing have initiated a paradigm shift in TE, by enabling precise control in various parameters such as cell distribution, porosity (better nutrient availability), and structural stability [1]. With these abilities, AM methods have immense potential to fabricate complex TE constructs and devise patient specific TE approaches [2]. However, it still has to be ensured that the biopolymer hydrogels employed in AM methods fulfilled the previously mentioned criteria.

Extrusion printing method has gained a lot of interest, as large constructs with clinically relevant dimensions with high cell densities can be easily fabricated, compared to other AM methods. In extrusion (bio-) printing, a hydrogel-cell suspension (called as “bioink” [3]) is extruded in a layer by layer fashion, according to a pre-set design to obtain a three-dimensional (3D) construct. Thus, the hydrogel component in the bioink has a vital role as it has to support the encapsulated cells, enable extrusion printing, and maintain its shape fidelity. Depending on the hydrogel used, the freshly printed construct is required to be stabilized—usually by crosslinking the hydrogel, to maintain long-term shape fidelity. Among the naturally derived biopolymer hydrogels used in extrusion bioprinting, alginate-based hydrogels have been extensively used mainly owing to its biocompatible ionic crosslinking by divalent ions. A critical drawback of ionically crosslinked alginate is its limited long-term stability in physiological conditions, arising from loss of divalent (calcium) ions over time into surrounding media and exchange for monovalent cations [4]. This leads to eventual disintegration of the alginate constructs, thus limiting its long-term applicability for TE [5]. Covalent crosslinking of naturally derived hydrogels [6] or chemical modifications such as methacrylation [7,8], enables covalent crosslinking of molecular chains of the biopolymers—thus, the hydrogels can be irreversibly crosslinked to maintain structural integrity over a long term. However, covalent crosslinking usually employs toxic chemicals (e.g., glutaraldehyde), or high-energy light (ultra violet) that are detrimental for the encapsulated cells [9]. Hence, there exists a need to explore and develop new hydrogels that could circumvent challenges associated with cytocompatible crosslinking and long-term stability.

Gellan gum, an anionic bacterial exopolysaccharide secreted by *Sphingomonas paucimobilis* [10], consists of repeating units of D-glucose, D-glucuronic acid, D-glucose, and L-rhamnose [11,12]. Owing to its biocompatibility, non-toxicity, resistance to heat and acid stress, and optical (transparent gel) properties—GG had found broad applications in food industry as a thickener [13] and is used as a substitute for agar in microbiological studies [14]. Native form of GG contains acyl groups on glucose residues of the chains (referred as high acyl GG; HAGG) [10], which interfere with ion-bonding ability [15] and form soft-elastic hydrogels [12]. Commercially available GG is deacetylated by alkaline treatment during production (referred as low acyl GG; LAGG) and form hard-brittle hydrogels [12,16]. Both forms of GG chains exist as random coils at high temperatures in aqueous solutions, which upon cooling self-assemble to paired helical structures at a sol-gel transition temperature (~50 °C) [17]. These multiple helices, called as junction zones, exhibit weak gel characteristics [18]. Addition of multivalent cations leads to formation of bridges between the helices, thus aggregating them to form a stable gel. The strong bridge formation between the helices render, practically, irreversible gelation [17].

Due to its favorable properties such as biocompatibility, easy processability, and long-term stability, GG received a lot of attraction for TE applications. The intrinsic advantage that the GG can be crosslinked by low concentrations of cations (~5 mM Ca^2+^ [18], similar to physiological solutions) means that GG hydrogels, in comparison to alginate-based hydrogels can be expected to maintain a stable structure long term, as it is needed for TE applications [19]. Furthermore, ability to easily include additives—blends with other biopolymer [20,21], bioactive particles [17]—and ability for easy chemical modification [16,22] has expanded its applicability in TE. The majority of the reported TE applications employed deacylated GG (LAGG) [17]. Many studies have reported 3D printing and bioprinting of GG composites with alginate [21], methacrylated gelatin [23], fibrinogen [24], or chemically modified GG such as methacrylated [25] or RGD-peptide grafted GG [26]. These approaches, i.e., modification of GG are majorly aimed at improving cellular response of encapsulated cells and tuning various physical properties. Application of pure LAGG for 3D bioprinting has not been possible as the gelation temperature is too high (>42 °C) to create a cell suspension. Its hard-brittle properties upon cooling to physiological temperatures would not allow formation of a homogenous cell suspension. To address this challenge, in our approach to develop GG as a bioink for 3D bioprinting—as the degree of deacylation determines the gel properties, different blends of HAGG and LAGG were developed with the intention to tune their rheological properties such that cells can be included in the biomaterial ink prior to 3D bioprinting. A rheological evaluation of the blends was performed to assess their printability and shape fidelity was determined when the blends were printed using three different needle diameters (250, 410, and 840 µm). Afterwards, stability under cell culture conditions and mechanical properties of the 3D-printed constructs were investigated. Lastly, after screening various LAGG and HAGG blends based on their stability, viability and proliferation of mesenchymal stem cells (MSCs) in the selected GG blend was evaluated. To compare the effect of adding an extracelluar matrix (ECM) like biopolymer to the GG, more blends consisting of selected LAGG and HAGG with 1 and 3% gelatin were also developed, characterized and the LAGG/HAGG blend containing 1% gelatin was used for bioprinting MSCs.

## 2. Results and Discussion

Blends of high and low acyl GG were prepared to combine the rapid gelling properties of LAGG and the elastic nature of HAGG. The highest possible concentration of LAGG was found to be 3% (*w*/*v*) in double distilled water (dd H_2_O). Attempts to dissolve higher concentration LAGG did not yield a homogenous solution. To prepare the blends, HAGG powder in desired concentration was added to 3% solution of LAGG and mixed using a spatula until a homogenous mixture was attained. A maximum of 3% (*w*/*v*) of HAGG powder in 3% LAGG solution resulted in a homogenous mixture. Blends containing gelatin were prepared by adding respective amounts of gelatin powder to 3% LAGG solution that was maintained at 50 °C under constant stirring. Different blends consisting of LAGG, HAGG, and gelatin (shown in Table 1) were analyzed regarding rheological properties relevant for 3D printing.

### 2.1. Rheological Evaluation of the Blends

**Figure 1:**
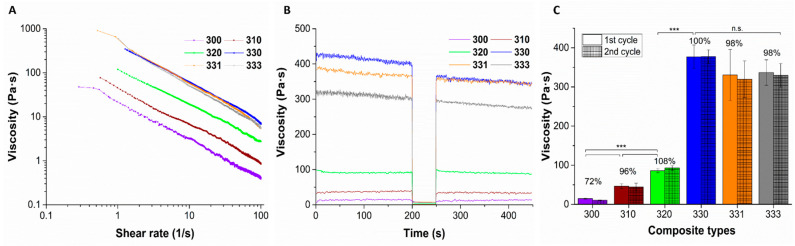
Representative plots of viscosity measurements indicating shear thinning (**A**) and shear recovery (**B**) behavior of different blends of LAGG, HAGG, and gelatin. Average viscosity of the blends measured when 2 cycles of low shear rate (1 s^−1^) was applied (**C**); *n* = 3, mean ± SD, *** *p* < 0.005, n.s. = non significant). Between these cycles, the blends were subjected to high shear rate (100 s^−1^) to mimic the printing process. Bold numbers represent the percentage viscosity recovery of the respective blends compared to the first cycle.

Viscosity of the blends was found to be increasing when concentration of HAGG added to LAGG was increased (measured at a shear rate of 1 s^−1^), owing to an increase in the total polymer content (Figure 1B,C). However, all the blends have shown shear thinning behavior (Figure 1A). Addition of 1% HAGG to 3% LAGG significantly increased the viscosity by approximately three times (viscosity of blends 300 and 310 was 14.8 ± 0.85 Pa·s and 46.30 ± 6.28 Pa·s, respectively). For the 320 blend, viscosity (86.20 ± 5.27 Pa·s) increase was approximately 1.8 times the viscosity of the 310 blend. Interestingly, further increment of HAGG concentration by 1% (*w*/*v*), i.e., for 330 blend, a significant increase in the viscosity (approximately 4.37 times as compared to 320 blend) was observed. Based on these observations, it can be postulated that the increase in viscosity of the blends with increase in concentration of HAGG, along with increase in total polymer content, presence of higher acyl groups in the blends probably contributed to this effect. Furthermore, these observations were in concurrence with the work of Bradbeer et al., where a similar increase in viscosity when HAGG concentration was increased in LAGG was observed [27]. In any case, the shear recovery of the LAGG had significantly improved with addition of HAGG, indicating that the blends of LAGG and HAGG could be 3D printed. The 3% LAGG gel (300 blend) had shown poor shear recovery (72%) and after extrusion through the needle, the strands had immediately lost their shape due to its low viscosity. Hence, 300 blend was rendered unprintable. Addition of 1% (*w*/*v*) gelatin to 330 blend had, however, resulted in reduction of viscosity (330.60 ± 65.16 Pa·s). Further increment of gelatin concentration (to 3% *w*/*v*) in 330 blend does not appear to alleviate this effect (336.90 ± 32.42 Pa·s). Lee et al. reported that addition of gelatin to GG reduced the intrinsic viscosity of the blend in comparison to pure GG [28]. Though the total polymer content was higher in 331 and 333 blends, reduction of viscosity can be probably attributed to the viscoelastic properties of gelatin.

### 2.2. Shape Fidelity and Volumetric Swelling Properties

**Figure 2:**
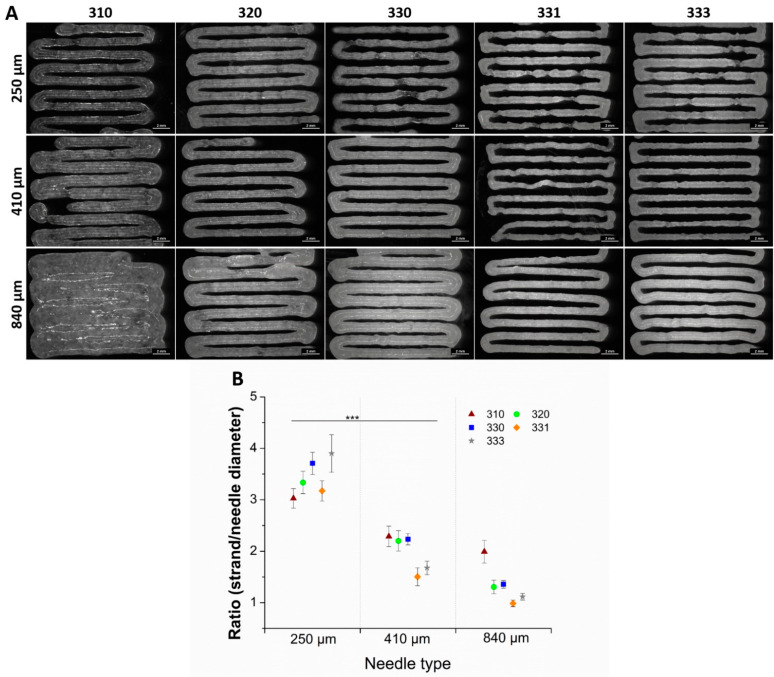
Optical images of single layer printed blends having 9 strands with a pre-set strand distance of 1.5 mm (**A**). Ratio of the measured strand diameter to needle diameter (scale bar = 2 mm) (**B**). Strand diameters are measured from the respective pictures at 20 different positions of single layer scaffold (*n* = 20, mean ± SD, *** *p* < 0.005).

Shape fidelity of the blends was determined by printing a single layer meander structure, using three different needles having an inner diameter of 250, 410, and 840 µm, and quantifying the strand diameters (Figure 2A). Increase in HAGG concentration in the blends resulted in better shape fidelity of the strands when printed with 410 and 810 µm needles (seen in the optical images, Figure 2A). The ratio of measured strand diameter after printing to ideal strand diameter, i.e., the needle diameter was calculated (Figure 2B). The calculated ratio was greater than one for all the blends, even though after printing with lowest possible pressure using three different needles—indicating that the blends exhibited Barus effect (die-swell effect) [29]. This effect was pronounced for the blends when printed with 250 µm needle and decreased when printed with needles with higher diameter. Also, increasing concentrations of HAGG in the blends had reduced the effect, especially when the blends were printed with 840 µm needles. After optical assessment and comparing the ratio of actual strand diameter to needle diameter, the shape fidelity of 330, 331, and 333 were found to be acceptable and these blends were used for further characterization studies.

**Figure 3:**
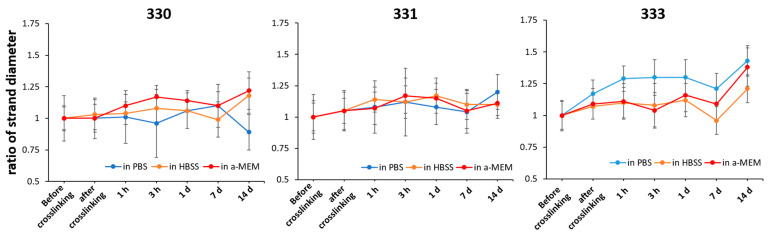
Change in the strand diameter of scaffolds over 14 days when incubated in PBS, HBSS, and α-MEM; expressed as a ratio of strand diameter at particular time point to strand diameter before crosslinking (*n* = 40, mean ± SD).

After the shape fidelity assessment, five layered scaffolds with five strands per layer of 330, 331, and 333 blends were 3D printed using a 410 µm needle. The printed scaffolds were stabilized by crosslinking them in 0.1 M CaCl_2_, followed by washing in phosphate buffer saline (PBS). The scaffolds were then incubated under cell culture conditions in three different solutions, namely phosphate buffer saline (PBS), Hanks balanced salt solution supplemented with calcium and magnesium (HBSS), and Minimum Essential Medium supplemented with 9% fetal calf serum (FCS), 1% l-glutamate, 100 U/mL penicillin, and 100 mg/mL streptomycin (α-MEM) to study their stability and strand swelling over a period of 14 days. As the 3D-printed (eventually bioprinted) GG scaffolds will be in cell culture media and conditions for long term, analysis of strand swelling and stability are deemed essential to estimate the eventual scaffold dimensions, probable effect on cells (after bioprinting), and handling of the constructs for analyses after the cell culture. Strand swelling for all the blends showed a similar trend when incubated in HBSS and α-MEM (Figure 3). For 330 and 331 scaffolds incubated in HBSS and α-MEM, an immediate increase in swelling was observed after 1 h, with further increase till 3 h, followed by decrease in swelling till 7 d and a steep increase at 14 d. For 333 scaffolds incubated in HBSS and α-MEM, an intermediate peak in swelling was observed after 1 d, followed by a decrease at 7 d and again showed highest swelling at 14 d. In the case of 331 and 333 scaffolds incubated in PBS, increase in gelatin concentration resulted in higher net swelling of the strands after 14 days. Scaffolds of 333 incubated in PBS showed highest swelling immediately after crosslinking and maintained a high swollen state till 14 days. In contrast, 330 scaffolds incubated in PBS showed reduction of strand diameter after 14 days—indicating the strands were probably degrading due to unavailability of cations and thus reduction in strand diameter was measured [30]. Though not significant, highest increase in the strand swelling was observed for 333 scaffolds incubated in all three types of solutions after 14 days. This increase in strand swelling can be attributed to presence of uncrosslinked gelatin. Kirchmajer et al. reported that swelling of crosslinked GG (by Ca^2+^ ions) and gelatin (by genipin) blends was lower than compared to individual gels [31]. Furthermore, a double network gel of GG-gelatin blends [32] could be expected to have lower swelling behavior.

### 2.3. Mechanical Properties of 3D-Printed Scaffolds

**Figure 4:**
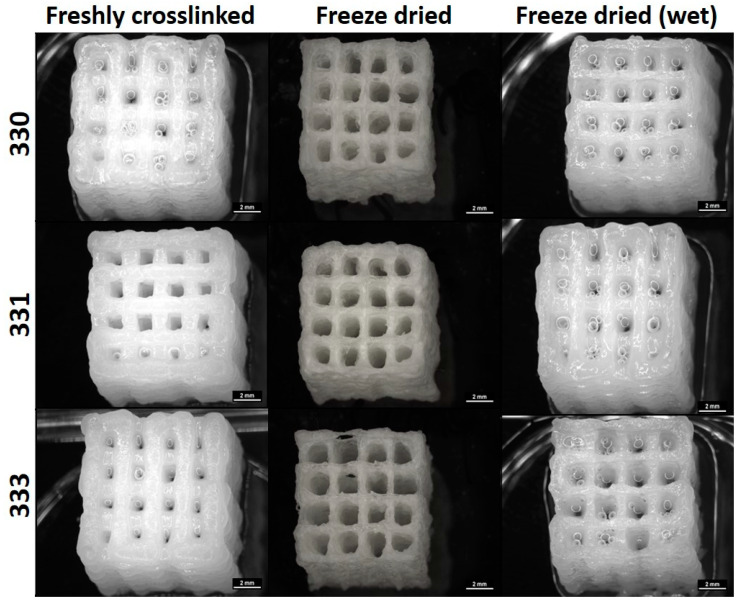
Images of 30 layered scaffolds obtained from a stereomicroscope taken immediately after crosslinking, freeze drying, and incubating the freeze dried scaffold in HBSS for 12 h (scale bar = 2 mm).

Mechanical properties were determined by performing uniaxial compression tests on the 3D-printed scaffolds having 30 layers (~10 × 10 × 10 mm^3^) with each layer having five strands printed with 410 µm needle (Figure 4). After crosslinking, a group of scaffolds were freeze dried and were also analyzed for their mechanical properties. Though not relevant for 3D bioprinting, freeze drying of polymeric materials can induce highly interconnected microporous structures in the scaffolds. Also, freeze drying can be deemed important to assess long-term storage possibilities. Such scaffolds can be used on demand as drug delivery systems, as substitute scaffolds for reconstructive surgeries, or for surface seeding of various cell types in vitro. For comparing the effect of processing conditions after 3D printing, compressive tests were performed after crosslinking the scaffolds with 0.1 M CaCl_2_, freeze drying, and after incubating the freeze-dried scaffolds in HBSS for 12 h (termed as “freeze dried wet”).

**Figure 5:**
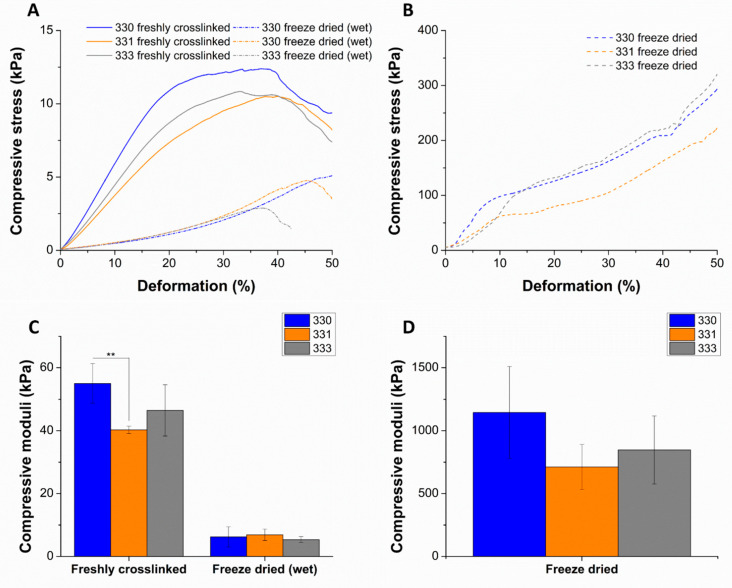
Representative plots of compressive stress vs. deformation as a result of uniaxial compression tests performed on scaffolds that were freshly crosslinked, freeze dried (wet) (**A**), and freeze dried (**B**). Compressive modulus determined from the slope of the curve till 10% deformation (**C**,**D**) (*n* = 4, mean ± SD, ** *p* < 0.01). The significant differences in the mechanical properties of freshly crosslinked, freeze dried (wet) scaffolds in comparison to freeze dried scaffolds can be noted with the scale of y-axis in the plots.

For freshly crosslinked and freeze-dried wet scaffolds, stress-deformation (strain) curves exhibited a typical brittle gel behavior where linear increase of stress was measured up to 40% deformation (pre-yield elasticity), followed by a non-linear regime (most likely due to irreversible disintegration of the scaffolds; Figure 5A). Freeze dried scaffolds showed a steep increase in the slope of curve till approximately 10% of deformation, followed by decrease in the slope till ~40% deformation; after which the slope appears to increase again (Figure 5B). The steep slope during the initial deformation period of the freeze-dried scaffolds can be attributed to the brittle nature of the scaffolds attained due to freeze drying of the polymeric material in the scaffolds. After around 10% deformation, the decrease in the slope must be a result of the polymer occupying the pores present in the strands due to compression. After 40% deformation, due to loss of pore volume, the scaffolds must be highly compressed, resulting in increase of measured stress.

**Figure A1:**
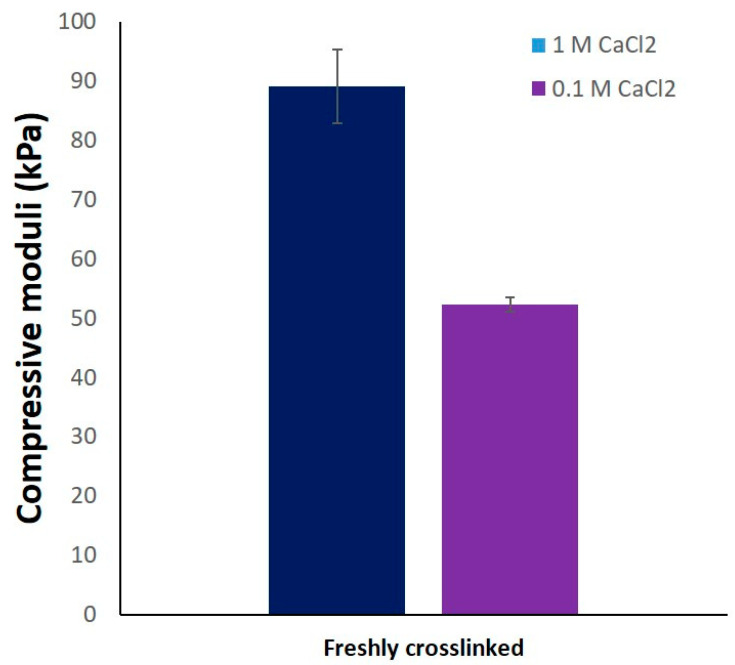
Compressive moduli of printed constructs of the composite 330 in dimensions of 10 × 10 × 10 mm^3^ and crosslinked with 1 M CaCl_2_ and 0.1 M CaCl_2_ (*n* = 3, mean ± SD).

Compressive stress measured till 10% deformation was used to determine the compressive moduli of the scaffolds in different conditions. Though not significant, average compressive modulus of 330 scaffolds after crosslinking (55.02 ± 6.26 kPa) and freeze drying (1144.70 ± 364.26 kPa) was higher compared to gelatin containing scaffolds. For gelatin containing scaffolds in freshly crosslinked and freeze dried state, an increase in average compressive modulus was observed with increase in the gelatin concentration of the scaffolds (Figure 5C,D). In case of freeze dried (wet) conditions, a significant drop of compressive modulus (~7–9 times) of all the scaffolds types was observed when compared to freshly crosslinked scaffolds. A similar observation was reported by Gupta et al., where the freeze-dried 3D-printed GG-gelatin scaffolds showed significantly lower mechanical strength in wet state even though the gelatin was chemically crosslinked [33]. This significant reduction in the mechanical strength of the freeze-dried wet scaffolds must be due to loss of calcium ions as the scaffolds were incubated for 12 h incubation in HBSS prior to compressive tests. The loss of calcium ions must have rendered low ionic crosslinking between the helical GG chains in the scaffold, resulting in low mechanical strength. An increase in the mechanical strength of 330 scaffolds when crosslinked with 1 M CaCl_2_ (Figure A1) confirms that divalent ion concentration proportionally determines the mechanical strength of 3D-printed GG scaffolds. In the work of Lee et al. to characterize mechanical properties of pure LAGG and blends containing HAGG, they reported that increase in HAGG concentration in LAGG significantly reduced the storage modulus of bulk gels and exhibited more elastic behavior [30]. Such an elastic behavior of the blends are desirable for bioprinting applications as the cells can be easily encapsulated in the blends before 3D printing.

### 2.4. Cell Viability of Mesenchymal Stem Cells in 3D Bioprinted

**Figure 6:**
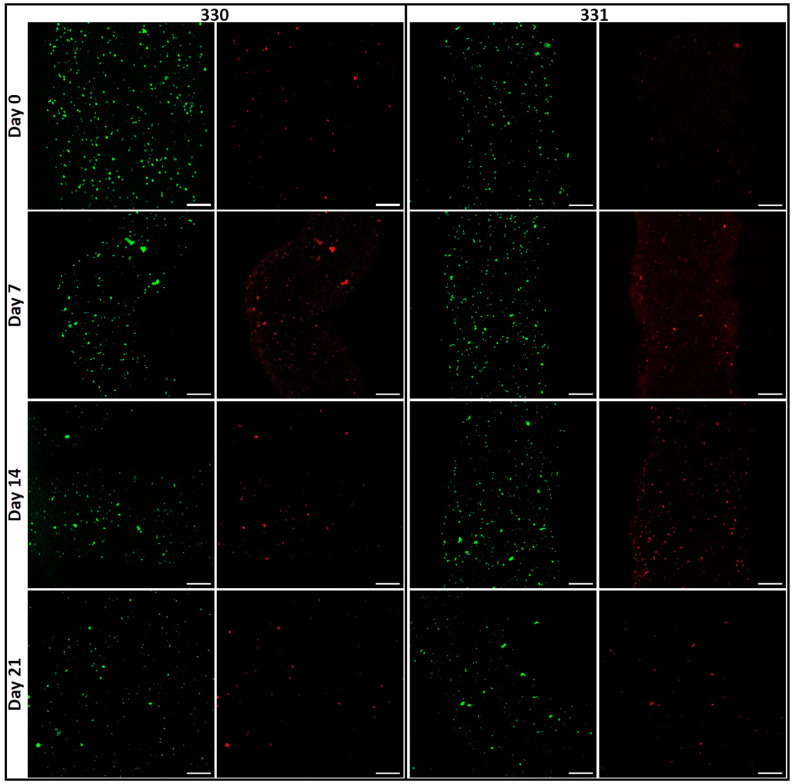
Images of viable (green; calcein) and dead (red, ethidium homodimer-1) cells acquired by cLSM immediately after bioprinting (day 0) till 21 days (scale bar = 200 µm).

**Figure 7:**
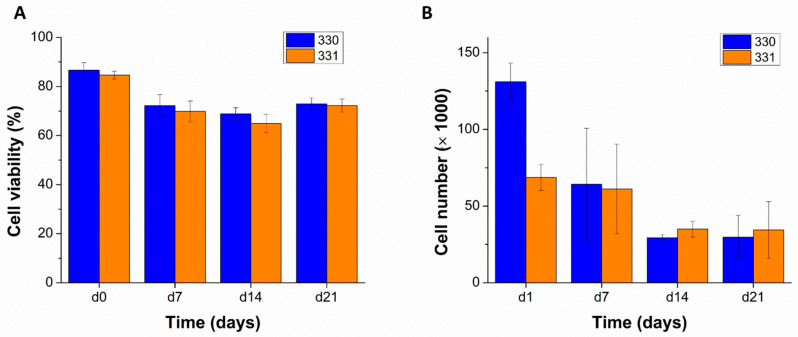
Total cell viability expressed as a ratio of viable cell to total cell numbers quantified from the cLSM images over 21 days, *n* = 5 ((**A**) *n* = 5, mean ± SD). Viable cell numbers quantified by biochemical assays determining LDH activities, over 21 days of MSC-laden scaffolds that were cultured in supplemented α-MEM ((**B**) *n* = 3, mean ± SD).

The blends 330 and 331 were used for performing bioprinting studies as these blends showed lowest swelling in α-MEM and were stable over a period of at least 14 days. To assess the suitability of these blends as bioinks—i.e., their ability to support cells when bioprinted—human telomerase reverse transcriptase (hTERT) expressing mesenchymal stem cells (MSCs) were mixed in them (at cell density of 5 × 10^6^ cells per gram of blend) and bioprinted in sterile conditions. As the hTERT-MSC’s can differentiate into osteogenic, chondrogenic, and adipogenic lineages [34]; cell-laden scaffolds can potentially be used for musculoskeletal tissue engineering. As an initial step in this direction, viability and proliferation of hTERT-MSC’s in GG scaffolds was evaluated by live/dead assay and by quantifying intracellular lactate dehydrogenase (LDH), respectively. Live/dead assay performed on the bioprinted scaffolds revealed a marginally higher cell viability in 330 blend (86.63 ± 3.10%) compared to 331 blend (84.62 ± 1.61%) immediately after completion of bioprinting (Figure 6 and Figure 7A). Later, decrease in cell viability till 14 days was observed for both the blends (i.e., 68.82 ± 2.52% and 64.91 ± 3.73% for 330 and 331 blends, respectively). However, an increase in cell viability was observed after 21 days compared to cell viability after 14 days (72.78 ± 2.54% and 72.21 ± 2.69% for 330 and 331 blends, respectively).

Interestingly, formation of cell clusters was observed already after seven days of culture (Figure 6), indicating that the cells in both blends preferred cell–cell contacts rather than to GG or gelatin in the constructs. It appears that the number of cell clusters are higher in 331 blend compared to 330 blend after 14 and 21 days of culture. As both LAGG and HAGG are devoid of any bioactive motifs, allowing integrin-based cell binding, cell spreading is not expected. In case of 331 blend, inclusion of 1% gelatin should have facilitated cell attachment and spreading as gelatin has abundant ECM proteins [35]. However, such an effect was not clearly visible in the live/dead assays of cell-laden scaffolds. A probable explanation for this observation could be loss of gelatin from the scaffolds in cell culture conditions (i.e., at 37 °C). In a related study of Ouyang et al., more than 70% of gelatin from scaffolds 3D printed with various bioinks (containing 5% (*w*/*w*) gelatin) was released within one day, when incubated in PBS at 37 °C [36].

Viable cell numbers in the bioprinted scaffolds, quantified by LDH activity measurements also showed a decrease till 14 days (Figure 7B). Low cell number measured in 331 scaffolds compared to 330 scaffolds at day one might be due to inhomogeneous mixing of the cells in the blend—as the cell suspension was mixed by hand using a spatula. To achieve uniform distribution of cells in the ink, a cell mixer unit [37] can be used prior to 3D bioprinting in the future. The measured cell numbers after 21 days of culture were similar to cell numbers after 14 days—supporting the observations made in live/dead assays. The decrease in cell viability of hTERT-MSCs in GG was in accordance to the work of Giglio et al. [38] where they used low acyl GG for cell encapsulation and reported a progressive decrease of cell viability from 78 to 68% from day one to day seven, respectively. Interestingly, higher cell viability of cells was reported in GG crosslinked with strontium ions compared to calcium ion crosslinked GG. Also, higher osteogenic differentiability of hTERT-MSCs in strontium crosslinked GG was observed, indicating that strontium ions facilitated cell viability and functionality of the encapsulated cells in GG. Different mechanical properties of strontium and calcium ion crosslinked GG gels are speculated to be the reason for the difference in encapsulated hTERT-MSCs response. In the current work, it can be speculated that lack (in case of 330) or insufficiency/inaccessibility (in case of 331) of cell adhesion sites and calcium crosslinking [38] resulted in round morphology of the encapsulated cells. Due to the uncharacteristic morphology of the hTERT MSCs, might have led to apoptosis over time.

Fabrication of complex functional structures to mimic native tissues usually require different mechanical properties within a single construct, spatial delivery of biological cues, or spatial organization of different cell types within a construct. Various methods of 3D extrusion printing [39] such as multi-material printing [40], core-shell printing [41], or solvent casting on 3D-printed constructs [42] can be used to realize such complex constructs. For example, osteochondral tissue substitutes require a gradient of mechanical strength (decreasing from osteon to chondral part), spatial separation of cell types, and delivery of growth factors (osteoblasts and chondrocytes; BMP-2 and TGF-β3 in osteon and chondral parts respectively) [43]. GG with its versatile properties, i.e., tunable mechanical properties by adjusting the concentrations of HAGG and LAGG or by chemical modifications [44] can be employed to fabricate scaffolds with gradient of mechanical properties. Furthermore, using multi-material 3D bioprinting, calcium phosphate cements (CPC) can be printed [45] with cell-laden GG to attain a single construct with materials suitable of osteochondral TE. This concept of multi-material bioprinting with CPC and plasma based bioink resulted in favorable cellular response including cell migration, proliferation and osteogenic differentiation of the encapsulated human preosteoblasts (hOB) and human dental pulp stem cells (hDPSC) [46]. Future work can be in the direction of preparing and characterizing plasma based bioink using GG so that explicit crosslinking (with CaCl_2_) can be avoided, as divalent ions in the cell culture media would be sufficient to maintain long-term stability. Further specific spatial mineralization in such constructs can be achieved by enzymatic methods, as was demonstrated by Douglas et al. [47].

## 3. Conclusions

With its versatile properties, easy processability, biocompatible crosslinking, and long-term stability, GG offers immense potential to be used by 3D extrusion printing and bioprinting for TE applications. As LAGG has brittle gel characteristics, its application for bioprinting would be limited. In our work, addition of soft-elastic HAGG to LAGG resulted in increase in viscosity and shear recovery of the blends, rendering them 3D printable. Also, very good shape fidelity of the blends confirms their 3D printability. Clinically relevant, volumetric constructs using these blends could be easily 3D printed. Low swelling properties and long-term stability of the constructs observed in α-MEM and favorable mechanical properties in wet state make the blends advantageous for bioprinting applications. In an initial study, 3D bioprinting of the blends with an immortalized MSC line showed high cell viability after one day, with a gradual decrease in cell number over 21 days of culture. More importantly, the cell-laden constructs maintained their shape and stability over 21 days in cell culture conditions. Combined with the observations in our work and the reported modifications of GG, there exists an immense potential of GG for 3D bioprinting applications. Furthermore, GG’s applicability can be significantly expanded by employing novel 3D extrusion printing methods like core-shell printing to cater specific demands in TE of various tissue types.

## 4. Materials and Methods

### 4.1. Preparation of GG-Gelatin Blends/Bioinks

Low acyl GG (LAGG) and high acyl GG (HAGG) were kindly gifted by CP Kelco (Atlanta, GA, USA). Gelatin from procine skin (Gelatin type A, 300 g Bloom) was obtained from Sigma-Aldrich (Darmstadt, Germany). All materials were γ-sterilized (25 kGy) in precursor powder form. LAGG was dissolved in Milli-Q water at nearly 100 °C under constant stirring until a clear solution was achieved. For gelatin containing composites, GG solution was cooled down to 50 °C, while stirring and gelatin is added and dissolved for 1 h followed by addition and mixing of HAGG in different concentrations with a spatula at room temperature until the composite is homogenously mixed. For cell culture studies, all materials were prepared under the sterile cell culture hood with sterile equipment.

### 4.2. Rheological Evaluation

All rheological tests were conducted using Rheotest 4.1 rheometer (Medingen, Germany), plate–plate geometry (diameter = 36.6 mm) with 0.1 mm gap at room temperature. To test whether materials show shear thinning behavior, shear sweep tests were carried out by measuring the viscosity with increasing the shear rate from 0 to 100 s^−1^ (with a shear rate increment of 0.1 s^−1^ per second). To demonstrate the effect of extrusion process on the pastes, viscosity recovery after applied shear was tested by applying shear rate of 1 s^−1^ for 200 s, then 100 s^−1^ for 50 s, finally 1 s^−1^ for 200 s again. The cycle was repeated twice and recovery after 1 to 10 s following the second shear cycle is divided by the initial average viscosity 1 to 10 s before shear, reported as % recovery as shown in Formula (1).

(1) $$\%\;\mathrm{Recovery}=\%\;\frac{\mathrm{Average}\;\mathrm{at}\;\left(1-10\;s\right)\;\mathrm{before}\;\mathrm{the}\;1\mathrm{st}\;\mathrm{cycle}}{\mathrm{Average}\;\mathrm{at}\;\left(1-10\;s\right)\;\mathrm{before}\;\mathrm{the}\;2\mathrm{nd}\;\mathrm{cycle}}$$

### 4.3. Assessment of Shape Fidelity and Swelling Properties

Using extrusion plotter (Bioscaffolder 3.1, GeSiM mbH, Radeburg, Germany), prepared blends were 3D printed with dosing needles having an inner diameter of 0.25, 0.41, or 0.84 mm (Globaco, Rödermark, Germany) to attain a single layer scaffold with 9 strand (with strand distance on 1.5 mm) as meander. Pictures of the scaffold immediately after 3D printing were taken using Leica M205 C stereo microscope equipped with a DFC295 camera (Leica Microsystems, Wetzlar, Germany). Strand diameters from the acquired images were measured using ImageJ (1.52 h, National Institutes of Health, Bethesda, MD, USA).

After determining the shape fidelity of the blends when printed with different needle types, swelling properties were studied on scaffolds printed using a 410 µm dosing needle and consisting of 5 layers with 5 strands per layer (with a strand distance of 2 mm). After printing, the scaffolds were immediately crosslinked for 10 min using 0.1 M calcium chloride (CaCl_2_, Carl Roth, Karlsruhe, Germany). Then, the scaffolds were incubated in PBS, HBSS (both from Gibco, Thermo Fisher Scientific, Waltham, MA, USA), and α-MEM supplemented with 9% fetal calf serum (FCS), 1% l-glutamate, 100 U/mL penicillin, and 100 mg/mL streptomycin (all from Biochrom, Berlin, Germany) at 37 °C and 5% CO_2_. Images of the scaffolds were acquired by the stereo microscope before and after crosslinking and after 1 h, 3 h, 1, 7 and 14 days of incubating in the respective solution/media. At each time point, the supernatant is completely removed and replaced with fresh solution/media. Strand diameters at 20 different positions on the scaffolds were measured using ImageJ. The ratio of the strand diameter at the specific time to ‘before crosslinking’ is then calculated. Based on their swelling ratio and stability in different solutions, suitable blends are selected for characterization of mechanical properties and bioprinting.

### 4.4. Characterization of Bulk Mechanical Properties by Compressive Testing

The selected blends for characterization of mechanical properties were 3D printed using a 410 µm dosing needle, to fabricate scaffolds having 30 layers (each layer having 5 strands with inter strand distance of 2 mm; dimensions of approximately 10 × 10 × 10 mm^3^). The scaffolds were then subjected to uniaxial compressive testing (using Zwick-Roell Z010 equipped with a 100 N and 10 kN load cell, Zwick-Roell, Ulm, Germany) at different processing states, i.e., after freshly crosslinked (with 0.1 M CaCl_2_), freeze dried, and freeze dried (wet state). For freeze drying, the scaffolds were frozen at −20 °C overnight, followed by freeze drying at −40 °C under vacuum overnight. Freeze-dried (wet) samples were incubated in HBSS overnight at 4 °C prior to the compressive tests. For compressive tests, a load cell of 100 N and a pre-load of 0.01 N was used for freshly crosslinked and freeze-dried (wet) samples, whereas a 10 kN load cell and a pre-load of 0.5 N was used for freeze-dried samples. A compression rate of 5% deformation of the initial height of the scaffold per minute was applied on the scaffolds. Compressive Modulus (E) was calculated from the standard force versus deformation (%) as the slope of the linear region at 0–10% deformation.

### 4.5. 3D Bioprinting of Cell-Laden Scaffolds

Selected blends, which showed lower swelling properties and stability in α-MEM (Section 4.3), were prepared (as described in Section 4.1) in sterile conditions. Human telomerase reverse transcriptase (hTERT) expressing mesenchymal stem cells (MSCs) [34] that were expanded in T75 flasks containing supplemented α-MEM medium were harvested and were mixed in the blends for 3D bioprinting of cell-laden scaffolds. Briefly, 5 × 10^6^ cells in 100 μL of supplemented α-MEM was added to the blends in sterile beaker and mixed carefully using a spatula. The cell-laden blend was then filled into 10 mL cartridges and bioprinted (as described in Section 4.3) in sterile conditions. The bioprinted scaffolds (having 5 layers with 5 strands per layer having a strand distance of 2 mm) were crosslinked with 0.1 M CaCl_2_ for 10 min, followed by washing with PBS to remove excess CaCl_2_ and addition of supplemented α-MEM. The cell-laden scaffolds were then cultured under cell culture conditions (37 °C and 5% CO_2_) for 21 days. Scaffolds were collected at each time point (i.e., after 1, 7, 14, and 21 days) by washing with HBSS, followed by freezing at −80 °C for biochemical analysis.

#### 4.5.1. Quantification of Cell Viability and Proliferation by Biochemical Analysis

Cell viability at each time point was analyzed and quantified by performing live/dead assays. Cell-laden scaffolds were incubated in supplemented α-MEM medium, containing calcein-AM/ethidium homodimer-1 (Invitrogen™ LIVE/DEAD™ Viability/Cytotoxicity Kit for mammalian cells, Thermo Fisher Scientific, Waltham, MA, USA) at 37 °C for 20 min. Followed by acquiring z-stack images of the scaffolds using a Leica TCS SP5 confocal laser scanning microscope (cLSM) (Leica Microsystems, Wetzlar, Germany) located in the MTZ Imaging Facility of Technische Universität Dresden (Dresden, Germany) (*n* = 5 per blend per time point). Excitation/emission wavelength for ethidium homodimer-1 and calcein AM was 528/617 and 495/515 nm, respectively. Number of live (stained by calcein-AM, green channel) and dead cells (stained by ethidium homodimer-1, red channel) were counted using cell counter plugin of the ImageJ. Cell viability was then determined by calculating the ratio of number of live cell to total number of cells (live and dead).

#### 4.5.2. Biochemical Analysis

Cell-laden scaffolds that were previously collected were thawed on ice, and homogenized (precellys^®^24 system, Peqlab, Erlangen, Germany) to disintegrated the scaffolds structure. Subsequently, cells were lysed at 30 min at 37 °C using 500 μL of lysis buffer (1% Triton-X-100; Merck, Darmstadt, Germany) and centrifuged at 4000 rpm for 5 min. To determine viable cells in scaffolds, LDH activity was measured by incubating the supernatants (lysates) with LDH substrate according to manufacturer’s instructions (CytoTox 96^®^ Non-Radioactive Cytotoxicity Assay; Promega, Madison, WI, USA). The reaction kinetics was determined measuring the absorbance till 5 min at wavelength of 490 nm using a microplate reader (Infinite^®^M200 PRO, Tecan, Männedorf, Switzerland). To quantify viable cell number, slope of absorbance vs. time of samples was correlated with the slope obtained for known cell numbers (served as calibration).

### 4.6. Statistical Analysis

Quantified results are presented as mean value ± standard deviation. All statistical analysis was carried out using OriginPro 8.5 (OriginLab, Northampton, MA, USA). One-way analysis of variance (ANOVA) was used to evaluate statistical significance at a level of *p* < 0.05.

## Figures and Tables

**Table 1:** Gellan Gum (LAGG and HAGG) composites without/with gelatin (Gel) and their abbreviations.

Blends/Bioinks	Abbreviations
3% LAGG only	300
3% LAGG + 1% HAGG	310
3% LAGG + 2% HAGG	320
3% LAGG + 3% HAGG	330
3% LAGG + 3% HAGG + 1% Gel	331
3% LAGG + 3% HAGG + 3% Gel	333

## Data Availability

The data supporting this article are shown as figures in the results and Appendix A. Raw datasets analyzed in the present study are available from the corresponding author upon reasonable request.
